# Supplementary material for: A high-resolution mRNA expression time course of embryonic development in zebrafish
Source: eLife. 2017 Nov 16;6:e30860. doi: 10.7554/eLife.30860 (PMC5690287; doi:10.7554/eLife.30860)
Supplement: Supplementary file 6. [file elife-30860-supp6.zip › biolayout-clusters-files/Cluster141-genes.html]

Cluster141


# Cluster141: Genes

| Ensembl ID | Gene Name | Chr | Start | End | Biotype |
| --- | --- | --- | --- | --- | --- |
| ENSDARG00000055078 | chac2 | 12 | 23934781 | 23939139 | protein\_coding |
| ENSDARG00000004260 | cul5b | 21 | 22087999 | 22106544 | protein\_coding |
| ENSDARG00000061214 | lipin2 | 2 | 30976818 | 31000975 | protein\_coding |
| ENSDARG00000039757 | mcfd2 | 13 | 8351615 | 8360837 | protein\_coding |
| ENSDARG00000034826 | park7 | 11 | 40847600 | 40861923 | protein\_coding |
| ENSDARG00000078745 | pnpla8 | 4 | 13627043 | 13644767 | protein\_coding |
| ENSDARG00000026484 | rab15 | 20 | 28880024 | 28898120 | protein\_coding |
| ENSDARG00000089458 | rp1l1a | 17 | 5611512 | 5665024 | protein\_coding |
| ENSDARG00000033437 | rps6ka1 | 17 | 27045901 | 27153154 | protein\_coding |
